# Development Trends and Perspectives of Future Sensors and MEMS/NEMS

**DOI:** 10.3390/mi11010007

**Published:** 2019-12-18

**Authors:** Jianxiong Zhu, Xinmiao Liu, Qiongfeng Shi, Tianyiyi He, Zhongda Sun, Xinge Guo, Weixin Liu, Othman Bin Sulaiman, Bowei Dong, Chengkuo Lee

**Affiliations:** 1 Department of Electrical and Computer Engineering, National University of Singapore, Singapore 117583, Singapore; elezhuj@nus.edu.sg (J.Z.); liu.xinmiao@nus.edu.sg (X.L.); eleshiq@nus.edu.sg (Q.S.); tianyiyi@u.nus.edu (T.H.); e0320823@u.nus.edu (Z.S.); guoxg@u.nus.edu (X.G.); elelwei@nus.edu.sg (W.L.); eleos@nus.edu.sg (O.B.S.); eledbo@nus.edu.sg (B.D.); 2 Center for Intelligent Sensors and MEMS (CISM), National University of Singapore, Singapore 117608, Singapore; 3 Hybrid-Integrated Flexible (Stretchable) Electronic Systems Program, National University of Singapore, Singapore 117608, Singapore; 4 NUS Suzhou Research Institute (NUSRI), Suzhou Industrial Park, Suzhou 215123, China; 5 NUS Graduate School for Integrative Science and Engineering (NGS), National University of Singapore, Singapore 119077, Singapore

**Keywords:** MEMS sensor, zero-power sensor, flexible sensor, human-machine interface, machine learning, artificial intelligence

## Abstract

With the fast development of the fifth-generation cellular network technology (5G), the future sensors and microelectromechanical systems (MEMS)/nanoelectromechanical systems (NEMS) are presenting a more and more critical role to provide information in our daily life. This review paper introduces the development trends and perspectives of the future sensors and MEMS/NEMS. Starting from the issues of the MEMS fabrication, we introduced typical MEMS sensors for their applications in the Internet of Things (IoTs), such as MEMS physical sensor, MEMS acoustic sensor, and MEMS gas sensor. Toward the trends in intelligence and less power consumption, MEMS components including MEMS/NEMS switch, piezoelectric micromachined ultrasonic transducer (PMUT), and MEMS energy harvesting were investigated to assist the future sensors, such as event-based or almost zero-power. Furthermore, MEMS rigid substrate toward NEMS flexible-based for flexibility and interface was discussed as another important development trend for next-generation wearable or multi-functional sensors. Around the issues about the big data and human-machine realization for human beings’ manipulation, artificial intelligence (AI) and virtual reality (VR) technologies were finally realized using sensor nodes and its wave identification as future trends for various scenarios.

## 1. Introduction

The microelectromechanical systems (MEMS) is the integration of electrical and mechanical components at the nanoscale and microscale dimensions [1,2,3,4]. MEMS and Nanotechnologies are commonly assumed as one of the most impressive topics due to their potential applications in both industrial and consumer areas [5,6,7,8,9]. MEMS are made of components between 1–100 µm in size, and MEMS sensors are generally defined as the range in size from 20 µm–1 mm. MEMS mainly evolve from the semiconductor manufacturing technology, i.e., the complementary metal-oxide-semiconductor (CMOS) process. For example, the basic techniques contain the deposition of material layers, patterning by photolithography, and etching for the device layout. MEMS manufacturing technologies are divided into surface micromachine and bulk micromachine. Surface micromachining uses the layers deposition on the surface of a substrate rather than using the substrate itself, whereas the bulk micromachining is the oldest paradigm of the silicon-based MEMS for building the micro-mechanical structures.

To identify parameter changes in the environments, the MEMS/NEMS sensors were developed by measuring its mechanical, magnetic, chemical, optical, acoustic, thermal, or electromagnetic information [10,11,12]. During the mid-1950s, researchers started to investigate the MEMS technology for a transistor. After that, a wide range of commercial devices based on the MEMS sensors was produced, such as inkjet printers, MEMS microphones, MEMS accelerometers, MEMS gyroscopes, MEMS/NEMS pressure sensors, display sensors, MEMS/NEMS switches, and MEMS/NEMS biosensors [13,14,15,16]. The mechanisms of the MEMS/NEMS sensing contain the piezoresistive method, the piezoelectric method, the capacitive method, the electromagnetic method, the thermal electrical method, etc. Among different kinds of MEMS-based sensors, the advantages of the silicon sensors involve their small size, high signal-to-noise ratio, low hysteresis, ability to work in an extreme environment, and high repeatability in their fabrication. Due to these advantages, the conventional MEMS sensors were widely applied to automation, aeronautics, consumer electronics, defense, industrial manufacture, medical equipment, life science, and telecommunication [17,18,19]. However, towards the new era, the MEMS sensors encounter new challenges in terms of their intelligence and power supply during their operation, such as the challenges of the flexibility for wearable applications, friendly and mutual interaction capability in the human-machine interface applications, and the intelligence required by the big data analysis. To reduce the power consumption, MEMS/NEMS switch and MEMS energy harvesting technology has been carried out to benefit MEMS sensors (called event-based sensor or accident-based sensor). With those MEMS/NEMS components, the MEMS sensors could operate for a long-time duration or in self-powered operation. As the improvement of the life quality for human beings, the wearable sensors and human-machine interfaces were identified as an important development direction for future sensors with enough flexible and multi-functional sensing. Meanwhile, combining with artificial intelligence (AI) and virtual reality (VR), the next-generation sensors will present a clear development trace and assist human beings interacting with other objects in a wide range of scenarios applications.

**Figure 1:**
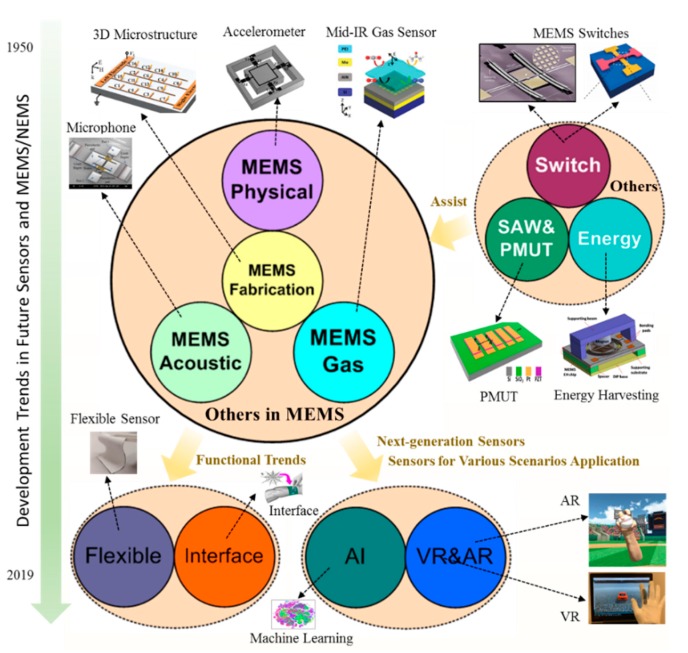
The development trends and perspectives of the future sensors and microelectromechanical systems (MEMS)/NEMS.

This review paper reports the development trends and perspectives of the future sensors and MEMS/NEMS. Starting from the MEMS fabrication technologies, the critical microfabrication technologies including soft lithography, transfer-printing, and 3D packaging technologies were discussed. Those technologies in MEMS sensors resulted in a fast-commercialization and better stabilities of the product. For an easy understanding of these kinds of MEMS/NEMS sensors, from the aspect of the operation frequency, we discussed MEMS physical sensors, MEMS acoustic sensors, and MEMS gas sensors, respectively. With the help of the mechanical vibration for the MEMS accelerometer and MEMS gyroscope, those kinds of MEMS physical sensors can be used as an energy harvesting device or the self-power sensor to identify its excitation acceleration. To reduce the power consumption during operation, the investigation of almost zero-power sensor or the wake-up sensors were raised up by researchers recently, which meant the sensors without any power consumption (called accident-based or event-based sensor). To overcome the issues of the weak-signal with high-level noise from MEMS acoustic sensors, machine learning technologies were recently adopted to identify the output of signals for the language control application. Aiming for the gas identification or health care diagnose, the current MEMS gas sensor was developed with small size, high sensitivity, low concentration detection, selectivity to different gases, and multi-gas sensing. With the assistance of 2D materials (graphene) or metal-organic frameworks (MOFs), low concentration and multi-gas detection were reported recently in the area of healthcare or disease diagnose. Toward the wearable sensing application, we observed the development trends from the conventional rigid silicon-based sensors to the flexible-based sensors, such as the functional materials textile in the wearable electronics application for our perceptions. The sensing functionalities of wearable electronics included but not limited to pressure, strain, electrophysiology, temperature, and blood oximetry. Furthermore, the rapid development of modern society has witnessed the increasing interaction between humans and machines, resulting in huge demands of smart human-machine interfaces. Around the big data and supersensitive sensing, MEMS/NEMS sensor using a machine learning method dramatically promotes the development of the next-generation intelligence sensing system. With the rapid development of information industry and human-machine interface technology in the fifth-generation cellular network technology (5G), virtual reality (VR) technology, and augmented reality (AR) technology have become a hot research topic when combined with MEMS/NEMS wearable devices to form a whole interactive system in 3D space. This kind of interactive system with future sensor nodes provides users with a more immersive experience and has the potential to be used in many application scenarios, such as sports training simulation, medical rehabilitation, entertainment, and so on (see in Figure 1).

## 2. MEMS Sensor in Internet of Things (IoTs) Application

With the increasing demand for the advancement of sensor technology to meet IoT requirements under 5G infrastructure, conventional MEMS sensors are facing a new round of evolution. Starting with the MEMS fabrication technology, which is fundamental for all sensors to be physically produced, new fabrication methods as described in Section 2.1 have been proposed and demonstrated in making those novel sensors. In terms of applications in IoT, three major types of MEMS sensors, which will be widely used almost everywhere, are then discussed in Section 2.2 Section 2.3 and Section 2.4 with orders of increasing working frequency.

### 2.1. MEMS Fabrication 

**Figure 2:**
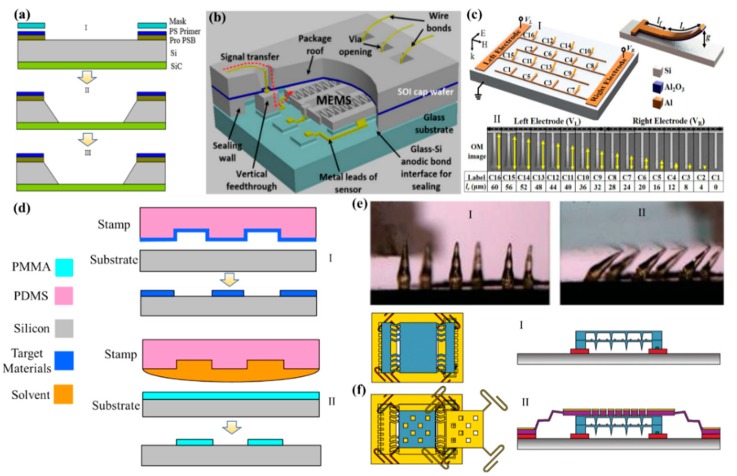
(**a**) Illustration of the typical fabrication of the MEMS sensor using bulk etching technology. (**b**) 3D view of a package with a wire bonding and a sealing technology. Adapted with permission from Torunbalci et al. [29]. (**c**) Schematics of a bandwidth tunable MEMS metamaterial supercell of microcantilever resonators with varying fixed and released lengths. Adapted with permission from Shih et al. [31]. (**d**) Schematic illustration of the two major methods involved in soft lithography. (**e**) A bendable microneedle array with a sharp tip and a lateral force was applied onto microneedle array. Adapted with permission from Wang et al. [35]. (**f**) Multi-layer MEMS structure was designed using direct laser writing technology, and the positioning and deposition processes were repeated. Adapted with permission from Reeves et al. [37].

MEMS fabrication is a key segment to produce MEMS sensors for IoTs applications. With the technologies including oxidation, ion implantation, chemical vapor deposition (CVD), metal sputtering, diffusion, exciting inventions were developed using those diversity process technologies [20,21,22,23,24]. A great number of impressive sensors were made with those technologies in the process of mass production. However, many critical issues in MEMS/NEMS fabrication still need to overcome, such as bulk etching, wire bonding, wafer-level packaging, and complex lithography [25,26,27,28]. To solve the conventional MEMS technology in the bulk etching with a rough surface, Figure 2a reports a typical MEMS fabrication using both potassium hydroxide (KOH) and Isopropyl alcohol (IPA) solution. The experiments showed good repeatability at a pressure of 5.0 MPa and with good surface roughness using the mixture solutions. To solve complicated wire bonding and its packaging [29,30], Figure 2b introduces a device using an anodic bonding and a vertical feed-through to eliminate the need for a sealing material. The packaging yield was experimentally verified to be above 95% under 15 MPa pressure. As for the 3D MEMS fabrication, an active metamaterial was adopted for a bandwidth tunable MEMS microcantilever application as shown in Figure 2c [31,32]. It was noted that the 3D MEMS structure consisted of microcantilever resonators of identical lengths with continuously varying release lengths. Lithography is a key process of MEMS fabrication. Along with the development of this technology, soft lithography using an elastomeric stamp was developed with its methods about the printing, molding, and embossing [33,34]. Figure 2d depicts two common methods using microcontact printing and nanostructures micro-molding. They demonstrated that the fabrication technology can be used for cell biology, lab-on a chip, and flexible electronics. As shown in Figure 2e, Wang et al. developed a bendable microneedle array using dual-assembled technology for skin penetration. They successfully demonstrated that the microneedles recovered to their original shape without any breakage with their dual-assembled fabrication technology [35,36]. Furthermore, to produce a much complex MEMS microstructures [37,38], 3D polymer microstructure using direct laser writing as shown in Figure 2f was developed to manipulate the 3D structure with the selective pattern for the mechanical elements. It claimed that the 3D MEMS microstructures had benefits for tunable resonant frequency characteristics and better sensing performance. Targeting the new fabrication techniques, new materials, and new structures, it is becoming clear that the future MEMS sensors are commercially viable and successful in the industrial and academic communities.

### 2.2. MEMS Physical Sensor

**Figure 3:**
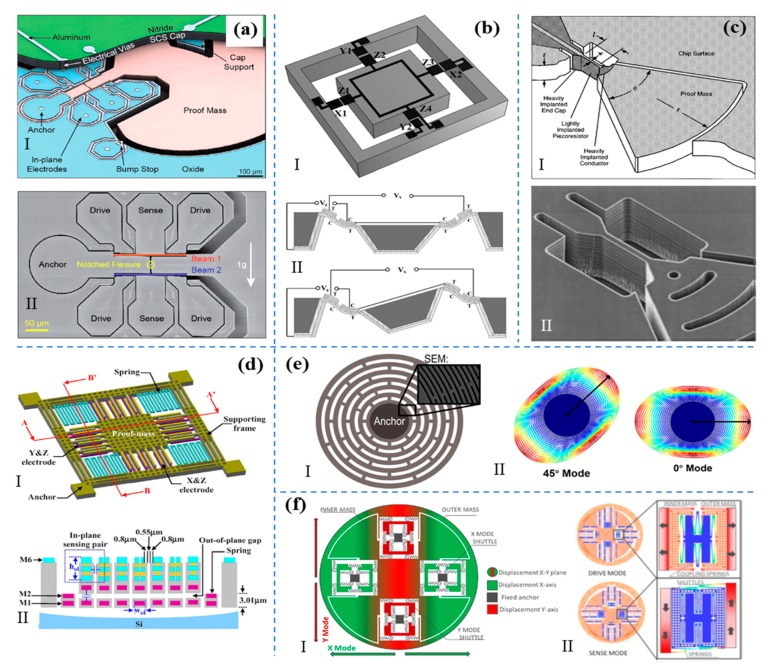
Various mechanisms of the accelerometers and gyroscopes. (**a**) The schematic of a resonant accelerometer. Adapted with permission from Shin et al. [39]. (**b**) The schematic of a piezoelectric accelerometer with tri-axis, and its operation states under vertical acceleration and lateral acceleration. Adapted with permission from Zou et al. [43]. (**c**) The schematic of a piezoresistive accelerometer. Adapted with permission from Partridge et al. [46]. (**d**) The schematic of a 3-axis capacitive accelerometer and its sectional view. Adapted with permission from Tsai et al. [49]. (**e**) The schematic of a resonant disk gyroscope. Adapted with permission from Nitzan et al. [52]. (**f**) The schematic of a vibratory gyroscope. Adapted with permission from Giner et al. [55].

MEMS physical sensor is one of the earlier studied components in the MEMS sensors, especially MEMS accelerometers and gyroscopes. Typically, the accelerometer can detect the deflections or stress deviations from an external acceleration through its electrical signals. Various MEMS accelerometers have been investigated by researchers based on different working principles, such as the resonant accelerometer [39,40,41,42], the piezoelectric accelerometer [43,44,45], the piezoresistive accelerometer [46,47,48], and the capacitive accelerometer [49,50,51], as shown in Figure 3a–d. Figure 3a shows the differential resonant beams accelerometer (DRBA) with high sensitivity, stability and at the same time high insensitivity to package stress and temperature variance proposed by Shin et al. [39]. When applied to an external acceleration, the stress caused by the proof mass will make these two beams to generate a differential signal of frequency and at the same time cancel out the influence of temperature and the package stress through their proximity. Besides, the resonant accelerometers based on double-ended tuning-fork (DETF) with micro-leverage and mode localization were also proposed [40,41,42]. Figure 3b depicts a piezoelectric mechanism for an accelerometer sensing in a 3-axis structure [43]. When an external acceleration was applied to the center proof mass, the connected MEMS beams in the corresponding deflection would generate a voltage output. Thus, the mathematical relationship between deflection and output voltage could be used to derive the applied accelerations. Beyond the piezoelectric sensing of acceleration, Figure 3c introduces the piezoresistive sensing principle for the accelerometer. The variance of the resistance was corresponding with the material deflection itself [46]. The weakness of this mechanism was that an external voltage was needed to drive the MEMS device during operation. The capacitive device was used often in the industrial area due to its long-term stability. Figure 3d shows the capacitive MEMS accelerometer design where the external acceleration applied to the system resulted in a variation of capacitance [49]. 

Gyroscope is the sensor aiming at measuring the angular variance or angular rate based on the Coriolis force. MEMS gyroscopes have been applied widely in various electronics and navigation systems and plenty of different structures have been proposed including the resonant gyroscope [52,53,54] and the vibratory gyroscope [55,56] as shown in Figure 3e–f. Figure 3e depicts a resonant disk gyroscope which has two resonant modes [52]. Through applied voltage, the disk will firstly vibrate at the drive mode or the 0° mode. When the device has an angular speed, the Coriolis effect will transfer energy to the secondary mode and effectively rotate the vibration pattern. The angular rate can be detected through the angular shift of this device. Figure 3f shows the vibratory gyroscope which generally has a drive frame, a Coriolis frame, and a detection frame [55]. Same as the resonant gyroscope, the drive frame will firstly keep vibrating in one direction through the electrostatic actuation by applying ac voltage, and at the same time drive the Coriolis frame while the detection frame keeps static. The force caused by the angular speed will make the detection frame move along another in-plane direction perpendicular to the driven direction and cause the capacitance variation. Furthermore, we can predict that the next generation MEMS accelerometer and gyroscope would achieve multi-direction sensing, higher sensitivity, and a lower noise floor [57,58,59,60,61]. What is more, endless research focused on a further decreasing the power consumption, system integration with IoTs, and new mechanisms of the MEMS accelerometer and gyroscope. In addition, as to the development of accelerometer, some designs that can tell the shake amplitudes and also the amount of energy of the movement have been put forward. These kinds of devices can be applied in IoTs for the application like mechanical monitoring through determining whether the vibration of a device is out of balance or is with an impact.

### 2.3. MEMS Acoustic Sensor

**Figure 4:**
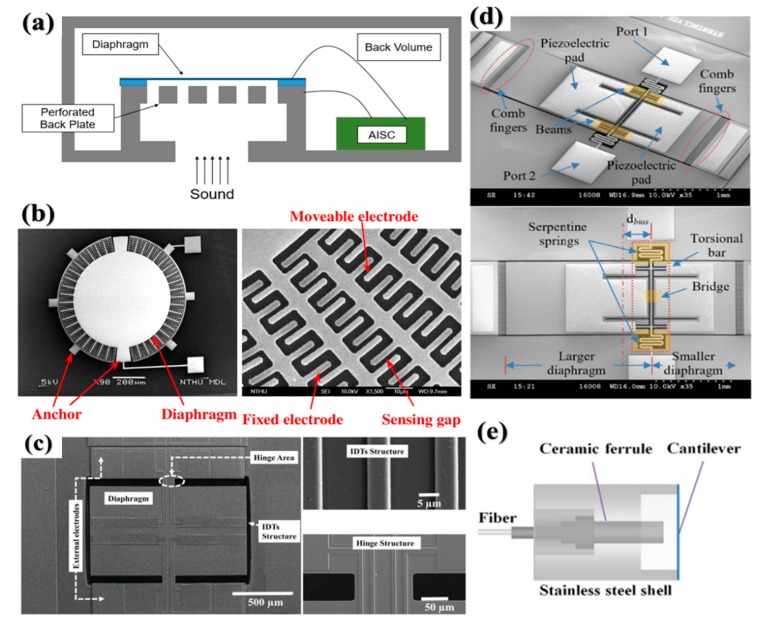
(**a**) Schematic of conventional capacitive MEMS microphone. (**b**) A no-back-plate capacitive microphone with interdigitated electrodes (IDTs) structure. Adapted with permission from Lo et al. [65]. (**c**) Bio-inspired piezoelectric microphone with a rectangular and a circular diaphragm. Adapted with permission from Rahaman et al. [69]. (**d**) Hybrid-mode microphone with a piezoelectric and a capacitive mechanism. Adapted with permission from Zhang et al. [71]. (**e**) Schematics of two optical microphones. Adapted with permission from Chen et al. [73].

MEMS acoustic sensor in which operation frequency ranges from 20–20,000 Hz is much higher than the typical MEMS physical sensor takes a big amount of MEMS market, especially its application in consumer electronics such as smartphones and smart speakers. The conventional MEMS acoustic sensor with working frequency in the audio range, aka microphone, consists of a flexible membrane and a back-plate with a bias voltage using a capacitive sensing mechanism as shown in Figure 4a [62]. Due to the limits of maximum signal level and sensitivity to environmental conditions, more sensing mechanisms have been investigated to improve acoustic sensor performance [63,64,65,66,67,68], which includes using back-plate-less design to minimize air damping, using piezoelectric sensing to achieve low power directional detection and using optical sensing to work at extreme environmental conditions, etc. Lo et al. proposed a no-back-plate structure that had 42 pairs of planar interdigitated electrodes (IDTs) in the same plane of the diaphragm as shown in Figure 4b [65]. The high-performance sensing was from the out-of-plane capacitance change due to no back-plate in a linear output and a less damping noise. The piezoelectric mechanism in acoustic sensing was the other impressive topic because of the less power consumption. As shown in Figure 4c, a bio-inspired piezoelectric microphone was reported for sound detection [69]. The IDT structures were placed on the top of the aluminum nitride (AlN) film to utilize *d*_33_ mode to identify the acoustic signal. The benefits of such design were directional sound detection, a lower noise floor, and no bias voltage. Furthermore, an optimization design was reported by Ishfaque et al. with a circular diaphragm [70]. To improve its acoustic electrical response, Zhang et al. introduced a combination of the piezoelectric and the capacitive sensing mechanisms for acoustic sensing as shown in Figure 4d [71]. It claimed that the asymmetric structure broadened its frequency response range and the later an improved cross design has achieved spatial sound detection capability [72]. Optical MEMS microphone gained increasing attention due to its extra high sensitivity, broadband frequency response, high signal-to-noise ratio, and immunity to electromagnetic interference. Figure 4e depicted a MEMS cantilever based optical microphone designs with high resolution and wide dynamic range [73]. The Fabry-Parot cavity formed between fiber tip and cantilever will change its length under acoustic waves and be measured by fast demodulated white-light interferometry, then it will be converted into electrical signals. In more advanced sensing mechanisms, direct coupling between acoustic waves and light are performed to achieve high sensitivity and low noise detection [74,75]. Table 1 has given a comparison among MEMS microphone types. In recent years, MEMS piezoelectric microphone has been commercialized and gradually taking up market shares especially in consumer electronics due to its outstanding low power consumption that allows constant standby. Moreover, to solve the application in the extreme environment condition and to keep a high precision measurement, optical microphones were also introduced for non-destructive testing operation around the ultrasound range. As the new technology development in 5G, we can predict that the future commercial MEMS microphone would be more compact and consume almost zero-power in the integration of the IoTs applications.

### 2.4. MEMS Gas Sensor

**Figure 5:**
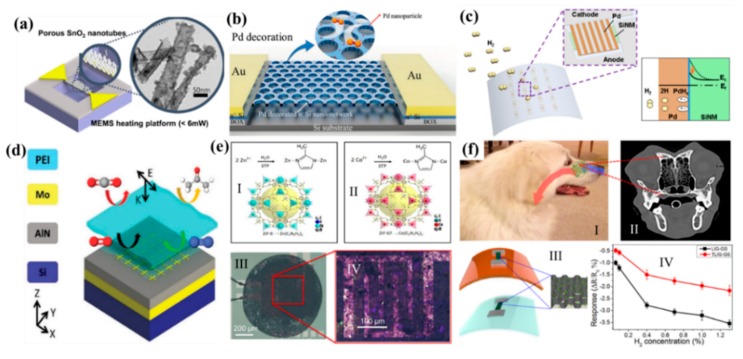
(**a**) Scheme of a localization synthesis of ZnO nanowire and SnO_2_ on the MEMS microheater. Adapted with permission from Cho et al. [78]. (**b**) Schematic of the Pd-decorated Si nanomesh H_2_ sensor. Adapted with permission from Gao et al. [82]. (**c**) Graphical illustrations of a flexible Pd/Si nanomembrane gas sensor, and an energy band diagram demonstrating the H_2_ sensing mechanism with the Schottky barrier. Adapted with permission from Cho et al. [84]. (**d**) Complementary metal-oxide-semiconductor (CMOS) platform for CO_2_ sensing in mid-IR. Adapted with permission from Hasa et al. [85]. (**e**) Image of the deposition of zeolitic imidazolate framework (ZIF)-8 nanocrystals solution by a drop coating. Adapted with permission from Matatagui et al. [87]. (**f**) Biomimic laser-induced graphene (LIG) gas sensor. Adapted with permission from Zhu et al. [89].

MEMS gas sensor is commonly used to identify the gas quality in all kinds of environments. The future MEMS gas sensor is aiming at a small size, high sensitivity, low concentration sensing, selectivity to different gases, sensors array, and multi-gas sensing [76,77]. Different kinds of metal oxide materials were used commonly in the MEMS gas sensor. The mechanism of this kind of gas sensor was from the band-gap energy which resulted in the resistance change. As shown in Figure 5a, Cho et al. reported a Tin oxide (SnO_2_) MEMS gas sensor with a localized synthesis of SnO_2_ nanotubes on the suspended microheater [78]. It was said that a very low power for their operations (<6 mW) was achieved in their design. The other research group carried out similar researches using different materials, such as Tungsten trioxide (WO_3_), and Zinc ferrite (ZnFe_2_O_4_) for various MEMS gas sensors [79,80,81]. However, the weakness of those materials was that the heat source is needed in their MEMS platform. To solve the MEMS gas sensing at room temperature, metal particles were reported for gas sensing with an advantage in good selectivity than metal oxide [82,83]. Gao et al. developed silicon-based nanosphere lithography for hydrogen (H_2_) sensing as shown in Figure 5b. The nanomesh structure with palladium (Pd) nanoparticles greatly enhanced the sensitivity of H_2_ sensing of 25% in 0.8% concentration. As to the enhanced sensitivity for the MEMS gas sensor, Cho et al. reported a high-performance and low-power flexible Schottky diode hydrogen sensor as shown in Figure 5c [84]. It demonstrated that the top-down fabrication process showed excellent H_2_ sensitivity (ΔI/I_0_ > 700 at 0.5% H_2_ concentrations) and fast response time (22 s) at room temperature (RT). The biggest advantage of waveguide gas sensing is the response time and sensitivity [85,86]. Take the mechanism using mid-Infrared radiation (IR) for example, Dihan et al. reported a carbon dioxide (CO_2_) gas sensor for 40 ppm detection at ambient temperature as shown in Figure 5d. The mechanism of the sensing was based on the infrared response with a gas-selective-trapping polymer for enhanced absorption in the mid-IR spectra. In a short time, Chang et al. reported a surface-enhancement infrared absorption for a detection limit of 20 ppm to CO_2_ [86]. To sense gas with a much lower concentration, nanostructures of zeolitic imidazolate frameworks (ZIF-8 and ZIF-67) [87,88] were developed as shown in Figure 5e. They said that the sensors can detect concentrations as low as 10 ppm of toluene, ethanol, carbon monoxide (CO), H_2_ and nitrogen dioxide (NO_2_), showing a large resistance change. In addition, 2D materials were the other development trend for the MEMS gas sensor [89,90], Zhu et al. reported a biomimetic gas sensor as shown in Figure 5f. They claimed that the biomimetic gas sensor demonstrated linear sensing to hydrogen. They also claimed that the biomimetic gas sensor demonstrated a much better gas sensing performance than CVD graphene-based gas sensors under the same dimension. The sensing performance comparison of those gas sensors was demonstrated as shown in Table 2.

## 3. MEMS Component in Future Sensor

Moving towards the future sensor, one of the most obvious trends is the formation of sensor networks with the individual sensor node. Under such a structure, future sensors as an integrated sensor system will undoubtedly face the challenges of low-power consumption, wireless transmission, and self-power generation. With the help of MEMS components such as MEMS switch, surface acoustic wave (SAW) and PMUT devices, and energy harvester, individual sensor nodes will be fully workable and connected to the networks.

### 3.1. MEMS Switch

**Figure 6:**
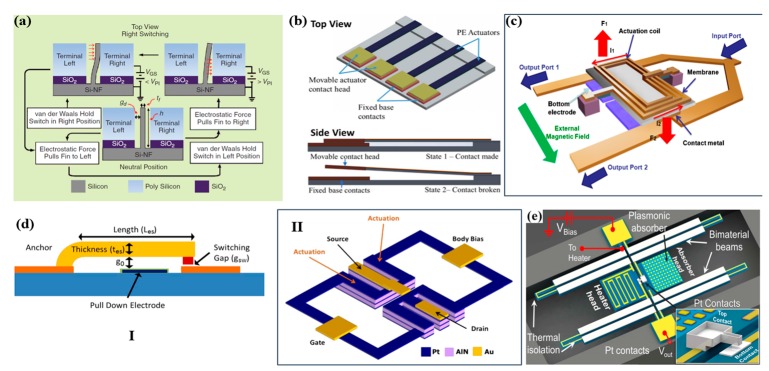
(**a**) Schematics of the switching behavior of the Si fin. Van der Waals force will hold the fin in contact position even after the electrostatic force. Adapted with permission from Soon et al. [97]. (**b**) Structure of the piezoelectric actuators array and the two states of switching. Adapted with permission from Maharjan et al. [98]. (**c**) Schematic diagram of the proposed push-pull radio frequency (RF) MEMS switch. Adapted with permission from Cho et al. [100]. (**d**) Schematic representation of the piezoelectric mechanical transistor, showing the three-finger dual-beam design. Adapted with permission from Sinha et al. [104]. (**e**) Structure of the plasmonically-enhanced micromechanical photoswitches (PMP) and working principle with scanning electron microscope images with highlighted magnified views of the plasmonic absorber. Adapted with permission from Rajaram et al. [105].

Micro-electrical contacts or MEMS switches have a range of existing and potential applications with MEMS sensors. As compared to conventional field-effect transistors, MEMS switches consume less power, have better isolation and insertion loss [91,92,93]. The advantages of MEMS switches can be achieved due to the mechanical actuation which is physically opened or closed the circuit. There are four basic actuation principles, electrostatic actuation [94,95,96,97,98], electromagnetic actuation [99,100], piezoelectric actuation [101,102,103,104], and electrothermal actuation [105,106]. Four typical principles of MEMS switches include the electrostatic principle, electromagnetic principle, piezoelectric principle, thermoelectric principle. Soon et al. introduced a bidirectional silicon nanofin switch as shown in Figure 6a. The switch maintained its contact leveraging on van der Waals force which held the silicon with an on-hold bias (100 nA output). Afterward, a dynamic circuit topology based on piezoelectric switches was reported by Maharjan et al. as shown in Figure 6b. They have claimed that the piezoelectric switches have the possibility to replace components as they can exhibit better failure tolerances and the ability to control multiple loads by interchanging the circuits as well as interchanging components to change the circuit configuration. Figure 6c reports a push-pull-type single pole double throw (SPDT) switch that achieved low power and low voltage operations by utilizing both electromagnetic and electrostatic forces. An electromagnetic force which can be produced by very low voltage is used to actuate the suspended membrane while the electrostatic force is used to maintain its state to reduce the power consumption. By using such configuration, it can achieve an insertion loss of −0.16 dB at 2 GHz and signal isolation of −54 dB. Figure 6d depicts a body-biased AlN piezoelectric MEMS switches which functionalize as a low voltage logic component. To operate at a voltage swing of ±1.5 V at 100 Hz, two AlN switch elements were used to form the body-biased structure. Figure 6e reports of a device with a standby power consumption which is near-zero with a low false-alarm rate till a specific input signal is detected. They have investigated the stability issue of the device which is due to the trade-off of the sensitivity of the sensor and the false-alarm rate which is due to the bias voltage is higher than 85% of the pull-in voltage. They have also demonstrated high threshold tunability. By adjusting the contact gap through varying the voltage bias, it gives the possibility to fabricate multi-threshold which is spectral selective. Furthermore, Jiang et al. reported a nanoscale memory cell which was also compatible with existing silicon technology on a switch. They claimed that the written data could be read with a standard dynamic random-access memory sensing circuitry. Typical power consumption for MEMS sensors was generally quite high and it would not be able to survive long. Trigger-based sensor switches can be designed to overcome this issue [99,103,107,108]. It would stay dormant to continue monitoring for signals but at a lower sampling rate. In such a way, MEMS switches could increase the lifespan of the MEMS sensor as it utilized a small fraction of the power.

### 3.2. MEMS Surface Acoustic Wave (SAW) and Piezoelectric Micromachined Ultrasonic Transducer (PMUT)

**Figure 7:**
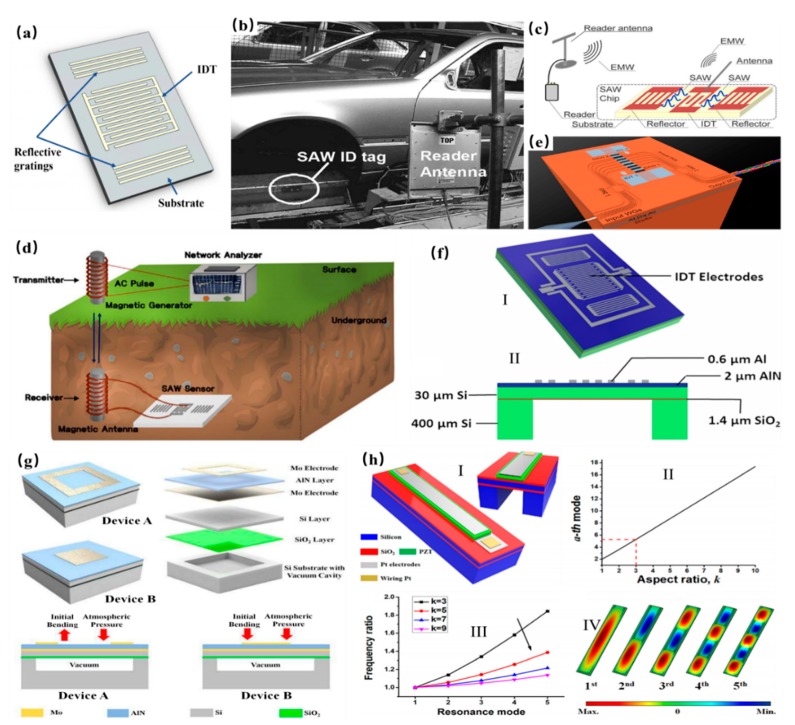
(**a**) A typical structure of a surface acoustic wave (SAW). (**b**) SAW ID tag application in a car assembly line. Adapted with permission from Plessky et al. [112]. (**c**) A wireless temperature monitoring system. Adapted with permission from Ma et al. [113]. (**d**) Wireless underground temperature sensor system with SAW. Adapted with permission from Kim et al. [114]. (**e**) Acoustically driven wavelength-division multiplexers using SAW. Adapted with permission from Crespo-Poveda et al. [115]. (**f**) Lamb wave sensor to decouple the viscosity and density of the liquid. Adapted with permission from Wang et al. [117]. (**g**) High-performance piezoelectric micromachined ultrasonic transducer (PMUT) with a zero-bending membrane. Adapted with permission from Wang et al. [118]. (**h**) Broadband PMUT based on a mode-merging. Adapted with permission from Wang et al. [119].

MEMS surface acoustic wave (SAW) devices play a significant role due to CMOS compatibility and easy fabrication. As the coming of the 5G network, SAW found its application in wireless communication and faster data transmission. As shown in Figure 7a [109], a kind of typical structure of the SAW device contained the interdigital transducer array (alternating current electrical signal into a periodical mechanical wave propagating on the piezoelectric substrate) and reflective gratings (the outward propagating acoustic waves within the cavity thereby minimizing the energy loss). Facing against effective communication, SAW tags had its advantage in a few meters reading distance with 2–3 orders of lower power consumption. Therefore, SAW tags were a suitable candidate in numerous applications, such as weapon tracking [110], the humidity sensing [111], and traffic control. Figure 7b shows a commercial SAW tag application in the automation car assembly line [112]. The identification code was written on the characteristics of reflected pulses from the SAW tag, with the number of codes reaching tens of thousands. Passive and wireless SAW sensors are competitive in the inhospitable and inaccessible environment for its robust and battery-free natures. Another application in the wireless measurement using the SAW device was an increasing need for remote monitoring and mutual communication. Figure 7c reports a wireless temperature sensing using SAW wireless mutual communication [113]. This wireless sensor had an antenna as the communication bridge for a temperature modulation. Moreover, Lee et.al proposed a sensor system to monitor underground temperature using the magnetic antennas as shown in Figure 7d [114]. It claimed that the reading distance reached up to 50 cm on the propagation line. Another promising area of SAW was acoustic-optic modulation as shown in Figure 7e [115]. The advantage of this technology was a high modulation frequency [116] and easy integration. In addition, viscosity and density are frequently used for label-free chemical detection, but they are difficult to be measured using traditional SAW sensors due to their joint effect on the shift of frequency. To address this issue, Wang et al. reported a novel SAW sensor based on AlN to decouple the effect of viscosity and density as shown in Figure 7g [117]. Lamb waves with higher-order modes were generated to interact with a liquid under the test. It was discovered that two unique modes only respond to viscosity and density, respectively, leading to different frequency shifts to decouple them. Thus, viscosity and density of the liquid under the test can be determined by a single device, showing promising potential for the clinical diagnosis and label-free chemical detection. Beyond the SAW technology, ultrasonic transducers are widely developed for applications in noninvasive imaging, range finding, non-destructive testing, non-contact communication, and sensing. Compared to the conventional ultrasound transducers, the PMUTs exhibited the attractive features of good impendence match with the soft medium, high transmission efficiency, and a low operation voltage. To achieve a maximum output performance, a zero-bending PMUT with the perfectly flat membrane (deflection of only 0.005%) was reported by utilizing the stress balance between AlN film, the frame-like top electrode and an encapsulated vacuum cavity as shown in Figure 7j [118]. A transmission sensitivity of 123 nm/V was achieved for the zero-bending device, about 450% higher than a reference device with non-zero deflection. Meanwhile, the broad frequency bandwidth of the ultrasound device was preferred in ultrasound imaging to achieve a higher resolution, but the conventional ultrasonic transducer and PMUTs normally had very limited bandwidth. To overcome this issue, a mode-merging PMUTs as a potential solution were investigated and developed [119,120,121]. As shown in Figure 7i, a wide frequency bandwidth was achieved using a rectangular diaphragm with a large length to width ratio [119]. With this design, few resonant modes can be excited with frequency quite close to each other. Then if the device was put in a highly damped medium like de-ionized water, these resonant modes merged and formed an ultra-wide frequency bandwidth (95% without matching layer). This ultra-wide frequency bandwidth can greatly improve the axial resolution of the ultrasound imaging without compromising its imaging depth.

### 3.3. MEMS Energy Harvesting

**Figure 8:**
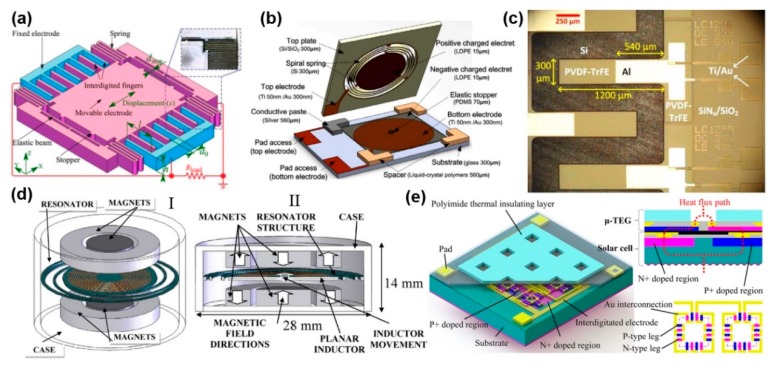
(**a**) The schematic of an electrostatic energy harvester. Adapted with permission from Lu et al. [134]. (**b**) An electret energy harvester. Adapted with permission from Tao et al. [135]. (**c**) The SEM with a false-color of a piezoelectric energy harvester. Adapted with permission from Toprak et al. [136]. (**d**) The schematic of an electromagnetic energy harvester. Adapted with permission from Sardini et al. [137]. (**e**) Hybrid energy harvester with the piezoelectric and electromagnetic mechanism. Adapted with permission from Yan et al. [138].

MEMS energy harvester is one of the MEMS components that can harvest various kinds of energy from the ambient environment and transfer it to electricity. Generally, the energy sources used for energy harvesting in the environment including mechanical energy, thermal energy, radiant energy, and biochemical energy [122,123,124,125]. Among all these energy sources, the mechanical energy was most suitable for the MEMS energy harvesting for sensor nodes due to its wide distribution. MEMS energy harvesting with different conversion methods had been investigated by many researchers, which contains the piezoelectric transduction [126,127,128], the electromagnetic induction [129,130] and the electrostatic transduction [131,132,133]. When applying an external vibration onto the electrostatic energy harvester as shown in Figure 8a, the distance or area of the comb structure changed with the capacitance variation [134]. Electret material-based energy harvesting is another common topic nowadays, whereas the electricity can be generated from the capacitance variation with a pre-charged electret material as shown in Figure 8b. It claimed that the optimization output power of 0.95 μW was achieved with a low resonance of 95 Hz [135]. Piezoelectric MEMS energy harvester was the research topic using the material piezoelectric effect which can convert the mechanical strain force from the structural vibration to electricity as shown in Figure 8c [136]. Maximum power output was calculated of 35.1 pW for a tip displacement of 500 µm from a 1200 µm × 300 µm cantilever. Figure 8d reports an electromagnetic energy harvester using Faraday’s law, which stated the phenomenon that current can be generated in a conductor within a magnetic field [137]. The hybrid energy harvesting has the advantage of higher energy output. As shown in Figure 8e, thermoelectric-photoelectric integrated energy harvester was put forward which can reach a higher energy density compared to traditional energy harvesters based on a single scavenging principle [138].

**Figure 9:**
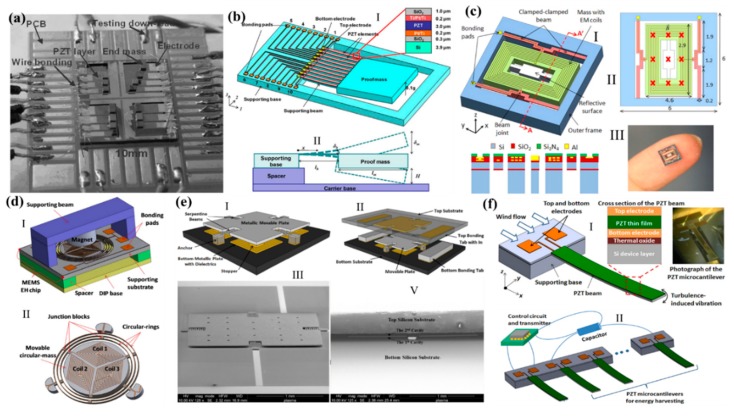
(**a**) A prototype of an array for a broaden frequency harvesting. Adapted with permission from Liu et al. [139]. (**b**) Schematic illustration of a piezoelectric energy harvesting for frequency-up in a constraint space. Adapted with permission from Liu et al. [143]. (**c**) Electromagnetic energy harvesting with several spring modes for a wide range of frequency energy harvesting. Adapted with permission from Liu et al. [144]. (**d**) Schematically drawing of the proposed 3D vibration electromagnetic device driven by a low-frequency for energy harvesting. Adapted with permission from Liu et al. [145]. (**e**) MEMS capacitive energy harvesters using stoppers for a low and a wide range of frequency. Adapted with permission from Zhu et al. [146]. (**f**) A wide range frequency vibration source of a microcantilever immersed in a wind flow for wind sensing and its energy harvesting. Adapted with permission from Liu et al. [149].

The biggest weakness of the MEMS energy harvester was featured with a fixed or a narrow operation frequency, whereas the frequency of an ambient vibration was floating in a wide range. To harvest energy at a broadening range, Liu et al. proposed the cantilevers array which tuned to the frequency and expanded the frequency bandwidth as shown in Figure 9a [139]. The prototype generator had a measurement performance of 3.98 mW of effective electrical power. The other method for the broadband frequency energy harvesting was the constraint of the space resulting in a dramatically nonlinear in the system [140,141,142,143]. As shown in Figure 9b [143], the piezoelectric MEMS energy harvesters were mounted on the carrier which has limited downward space and can act as a stopper to widen the operational bandwidth for this energy harvester. With this novel structure, this energy harvester can now have a broad bandwidth output power from 30 Hz to 47 Hz and 19.4 nW to 51.3 nW when vibrating at 1g. Furthermore, to harvest energy at an ultra-wide bandwidth frequency, the duffing stiffening of the clamped-clamped beam by three distributed resonances was introduced as shown in Figure 9c [144]. It claimed that the microfabricated device was able to broaden the operating bandwidth from 62.9 Hz to 383.7 Hz. After that, Figure 9d depicts a 3D dynamic behavior of the three vibration modes of 1285, 1470, and 1550 Hz [145]. The measurement of this MEMS structure achieved 0.444, 0.242, and 0.125 μW·cm^−3^ maximum power density at their vibration modes with an excitation acceleration of 1 *g*. Around the nonlinear components with MEMS stoppers [146,147,148], Zhu et al. developed the energy harvester using MEMS capacitor stoppers for frequency-up technology as shown in Figure 9e. It achieved energy harvesting with a limited amplitude and a very low frequency (much lower than its MEMS resonance frequency). To take advantage of the external wide range excitation [149], a flow sensing, and its energy harvesting were investigated as shown in Figure 9f. The output voltage and output power were measured as 18.1 mV and 3.3 nW at a flow velocity of 15.6 m/s, respectively.

## 4. MEMS/NEMS in Flexible and Interface

Flexible sensors have been a hot research topic in recent years due to its versatility and huge potential in health/medical applications. The flexible sensor is usually merged with a wearable sensor for its unique advantages. Other than just using the flexible sensor as a wearable device to achieve monitoring features, it can even be used as a human-machine interface to realize greater demands. In this section, these two topics will be specifically discussed.

### 4.1. Flexible Sensor

**Figure 10:**
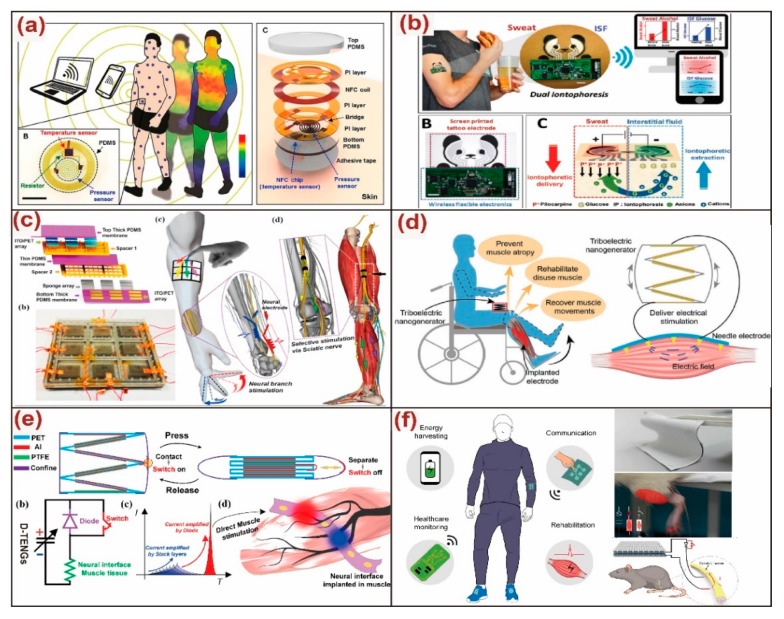
(**a**) A battery-free wireless pressure and temperature dual sensors. Adapted with permission from Han et al. [153]. (**b**) A wearable biosensor for simultaneously monitoring sweat and interstitial fluid. Adapted with permission from Kim et al. [154]. (**c**) A triboelectric nanogenerator (TENG)-based neuromodulator for peripheral nerve stimulation. Adapted with permission from Lee et al. [155]. (**d**) A self-powered TENG system for direct muscle stimulation. Adapted with permission from Wang et al. [156]. (**e**) A diode-amplified TENG for high-efficiency muscle stimulation. Adapted with permission from Wang et al. [157]. (**f**) A current-enhanced TENG textile system for healthcare monitoring and rehabilitation applications. Adapted with permission from He et al. [158].

Wearable electronics can be easily merged with human beings’ bodies to extend our perceptions. The sensing functionalities of wearable electronics included but not limited to pressure, strain, electrophysiology, temperature, blood oximetry, etc. [150,151]. With the aid of multiple devices across different anatomical locations, it is feasible to facilitate the development of the human body sensor network for applications from hospital care and clinical medicine to fitness and wellness tracking, awareness and cognitive state assessment, and human-machine interfaces [152]. Rapid developing in physical sensors with capabilities had demonstrated the growing significance and potential utility of these sensing platforms. Recently, Han et al. developed a battery-free wireless body sensor network that provided both the pressure and temperature sensing as shown in Figure 10a [153]. Each sensor contained a pressure-sensitive monocrystalline silicon layer and a miniaturized temperature sensor. This skin-like device can precisely measure local pressure and temperature as it was attached to the body. Biosensors that were in contrast with the physical sensors in terms of sensing mechanisms and designing techniques, was also vital for a comprehensive healthcare monitoring in daily life. Kim et al. reported a wearable biomonitoring device that enabled a simultaneous and yet independent analysis of two epidermal biofluids in a single device (Figure 10b) [154]. The dual biofluid sampling was realized through a sweat stimulation at an anode and an extraction of interstitial fluids at a cathode. Rehabilitation or disease treatment with wearable electronics had also become an emerging field in the past decades. Among them, the modulation of peripheral nerves was intensively investigated as a promising research field towards the next generation of neurotechnology. Lee et al. achieved an efficient nerve stimulation with a novel water/air hybrid triboelectric nanogenerator (TENG) as shown in Figure 10c [155]. The results demonstrated that the special waveform of the triboelectric output current was more efficient for the nerve stimulation with better linear control than a conventional biphasic square waveform. Recently, Wang et al. reported a self-powered system of a stacked-layer triboelectric nanogenerator and a multiple-channel epimysia electrode to directly stimulate muscles as shown in Figure 10d [156]. Although TENG provided a high voltage output, the current amplitude was quite low for achieving efficient muscle stimulation. To address the low-current issue of the direct muscle stimulation with a TENG, Wang et al. proposed a new general configuration of TENG device where a diode and mechanical switch was integrated together to amplify the current output and boosted up the current frequency to match with the resonant frequency of the motoneurons as demonstrated in Figure 10e [157]. However, these triboelectric devices were still bulky and rigid to fulfill the requirement of comfortability of wearable electronics. A flexible and soft textile-based TENG was then developed by He et al., and an instantaneous discharging approach of the diode-switch integration was adopted for current amplification as shown in Figure 10f [158]. The soft, flexible, and thin characteristics of the TENG textile enabled a moderate output under various operations even as it was randomly scrunched. Additionally, the enhanced current can efficiently stimulate rat muscle with a simple electrode, and the nerve stimulation with a small-size device was achieved as well. With the rapid development of recent self-powered technologies, wearable electronics are evolving towards self-sustainable systems to ultimately eliminate the requirement of external power supply [159,160]. 

### 4.2. Interface of Sensor

**Figure 11:**
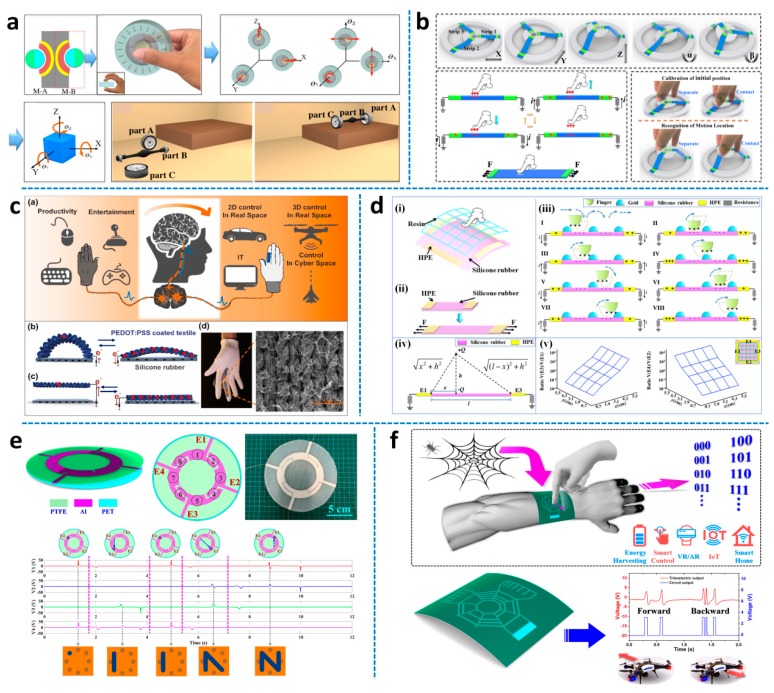
Triboelectric nanogenerator based human-machine interfaces. (**a**) Self-powered triboelectric based 3D-control sensor. Adapted with permission from Chen et al. [172]. (**b**) Multi-dimensional nano-manipulation terminal using strip sensors. Adapted with permission from Chen et al. [173]. (**c**) Conductive textile-based glove interface. Adapted with permission from He et al. [174]. (**d**) Flexible wearable patch for robotics manipulation. Adapted with permission from Chen et al. [175]. (**e**) Multi-functional and minimalist interface using a flexible wearable triboelectric patch. Adapted with permission from Shi et al. [177]. (**f**) Single-electrode bio-inspired spider-net-coding interface. Adapted with permission from Shi et al. [178].

The rapid development of modern society has witnessed the daily increasing intimacy between humans and machines, resulting in huge demands of smart human-machine interfaces [161]. TENG with the coupling effect of triboelectrification and electrostatic induction has been proven as a promising energy harvesting technology since its first invention in 2012 [162,163,164,165]. It has been extensively investigated to develop high-efficient energy harvesters, sensors, and actuators, with merits of high output performance, simple manufacturing, no material limitation, flexibility, good adaptation, and cost-effectiveness compared to other mechanisms. Meanwhile, triboelectric based human-machine interfaces have also been widely developed for various interacting applications [166,167,168,169,170,171], ranging from 2D to 3D, macro scale to micro scale, real space to cyberspace, daily control to industry manipulation. As shown in Figure 11a, a self-powered 3D-control sensor was presented. When the suspending sphere contacted with the bottom surface under human actuation, the generated charges on the contact area can be detected by the corresponding electrodes [172]. Thus, through signal analysis on the eight electrodes’ output, 3D force information (normal force and shear force with different directions) can be achieved and applied for the object attitude control with six degrees of freedom. The demonstration of parts assembly in 3D virtual space was successfully realized by using the sensor to control the position and rotation of different parts. Another multi-dimensional sensor was also developed using stretchable strip sensors for nanomanipulation as depicted in Figure 11b [173]. The device consisted of a fixed base, a mobile stage, and three symmetric strip sensors. After actuation, the position and rotation of the mobile stage (corresponding to object attitude) can be determined by finger contacting the same reference point of the three-strip sensors, since the different lengths of the strip sensor associated with different output ratios on its two electrodes. The 3D information of the mobile stage can then be used for the control of a nano-manipulator in a scanning electron microscope (SEM). Moving forward, flexible wearable interfaces will be of increasing importance to enable intuitive interactions between humans and machines [174,175,176]. Figure 11c shows an intuitive glove interface, with four textile-based sensors to achieve full interacting functionality [174]. Each sensor consisted of two parts, a polymer-coated conductive textile, and a silicone rubber layer. According to the design, different finger motions triggered a specific sensor to undergo contact and separation, thus inducing corresponding output signals. Using signal analysis, different finger gestures can be recognized and applied for various applications, such as wireless car/drone control, cursor control, gaming alphabet writing. A 2D flexible wearable patch and a 1D stretchable sensor were illustrated in Figure 11d, toward 3D robotics manipulation using the combined sensory information [175]. Through the integration of a grid structure on top, the flexible wearable patch detected not only the position of finger tapping but also the continuous finger sliding trace. Thus, by combining the in-plane sensory information from the patch with the out-of-plane sensory information from the 1D sensor, 3D manipulation of the robotic arm is successfully achieved for position, velocity, and trajectory control. Figure 11e depicts a minimalist triboelectric patch with only four sensing electrodes for multi-functional applications [177]. The patch was composed of three thin layers of polyethylene terephthalate (PET), Al, and polytetrafluoroethylene (PTFE). The layout of the four electrodes defined four individual points and four common points on the electrode area. When finger taps/slides on the common point, outputs were generated on two adjacent electrodes. Through leveraging the predefined points, position detection of both finger tapping and finger sliding can be achieved for writing interface, inputting interface, security code system, intuitive control, etc. A minimalist single-electrode interface based on bio-inspired spider-net-coding (BISNC) was presented in Figure 11f [178]. In the BISNC interface, the connected grating electrodes were introduced with information coding to achieve high usability and scalability. When finger slides across a specific direction, positive charges on finger induce output signal corresponding to the coding patterns on the single electrode. Thus, different directions can be decoded for broad applications including smart control and security coding system.

## 5. MEMS/NEMS vs. AI and Its VR and AR

When it comes to future sensors, artificial intelligence (AI) would unquestionably play a vital role in terms of “decoding” signals from the various sensor system and networks [179,180,181,182,183,184,185,186,187,188]. Furthermore, VR and AR will also rely much on novel sensors to provide better functionality such as immersive experience and feedback control. In this section, the focus will be made on how MEMS/NEMS will find its integration for the future.

### 5.1. MEMS vs AI

**Figure 12:**
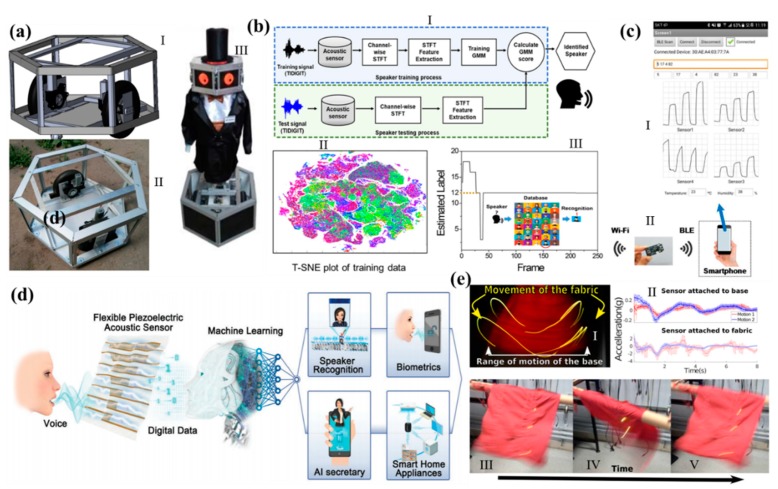
(**a**) Illustration of the dynamic neural network (DNN) for the performance of the low-resonant-frequency piezoelectric MEMS energy harvester. Adapted with permission from Nevlydov et al. [180]. (**b**) Visual representations of the gaussian mixture model (GMM)-based machine learning algorithm for the speaker recognition, and the dataset of 90% used for training data, and 10% for testing data by 2800 training data of 40 people. Adapted with permission from Han et al. [184]. (**c**) Raw data transmission of multiple sensors through Wi-Fi for data transmission. Adapted with permission from Suh et al. [185]. (**d**) Schematic illustration of the promising applications using flexible piezoelectric acoustic sensors in response to the speaker’s voice and the data were trained using a machine learning-based model. Adapted with permission from Jung et al. [186]. (**e**) Effects of motion on fabric, the motion signals from sensors were located on both a rigid base and loosely attached to the base via the fabric. Adapted with permission from Michael et al. [187].

Around the big data and supersensitive sensing, MEMS/NEMS sensor using machine learning promotes the development trend of the next generation intelligence system [179]. For example, an impressive AI concept sensor (called AIfES) with a completely configurable artificial neural network was developed. This sensor-related AI system can recognize handwriting and gestures, which successfully demonstrated the application of the next-generation sensors. Nevlydov et al. developed a robot using machine-learning methods as shown in Figure 12a [180]. They successfully adopted a three-axis MEMS gyroscope fixed to the robot body using various machine-learning methods for solving classification tasks. To enhance the vibration energy harvesting at low frequency, Nabavi et al. reported a piezoelectric MEMS vibration energy harvester at low (less than 200 Hz) resonant frequency based on the deep neural network [181]. This trained network integrated with the genetic algorithm to optimize the geometry of the energy harvester with the accuracy of the network in prediction above 90%. As to the area of gas sensing prediction, Esposito et al. introduced the dynamic neural network (DNN) method in a stochastic prediction of air pollutant concentrations to the chemical multisensory [182]. When it came to sound detection, Han et al. reported a platform using a machine learning-assist method for speaker recognition as shown in Figure 12b [184]. They claimed that the Gaussian Mixture Model (GMM) method in machine learning can reach up to an outstanding speaker recognition rate of 97.5% compared to the reference MEMS microphone. Furthermore, Suh et al. reported a fully integrated and portable semiconductor-type multi-gas sensing module for IoTs applications as shown in Figure 12c [185]. The module’s multiple gas sensing capability enabled selectivity enhancement by principal component analysis (PCA) method. The fabricated sensors showed decent response and recovery to the frequent heater on/off repetition for both portable and stationary applications. Furthermore, Jung et al. reported flexible piezoelectric acoustic sensors and machine learning for speech processing as shown in Figure 12d [186]. They claimed that smart sensors can provide personalized services. They further predicted the development trends using flexible sensors around flexible piezoelectric materials, machine learning algorithms, and speaker recognition. As to the more complex objects, Figure 12e reports wearable sensors on loose cloth [187,188]. They claimed that the multi-sensors mounted onto fabric showing significant differences than onto the rigid substrate. It predicted that a trade-off between additional information and intense motion can provide better accuracy than the conventional method for signal acquisition.

### 5.2. Toward VR and AR

**Figure 13:**
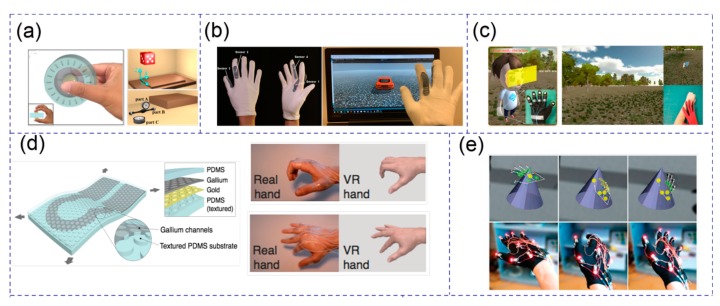
(**a**) A self-powered triboelectric based virtual reality (VR) 3D-control sensor. Adapted with permission from Chen et al. [172]. (**b**) A self-powered glove-based intuitive interface for diversified control applications in real/cyberspace. Adapted with permission from He et al. [174]. (**c**) A stretchable and transparent tactile sensor based on a hetero-contact microstructure for virtual human control. Adapted with permission from Liao et al. [190]. (**d**) Gallium-based thin films for wearable human motion sensors. Adapted with permission from Dejace et al. [191]. (**e**) A vibrotactile glove for identifying virtual 3D geometric shapes. Adapted with permission from Martinez et al. [192].

With the rapid development of the information industry and human-machine interface technology in recent years, VR technology and augmented reality (AR) technology have become a hot research topic when combined with MEMS/NEMS wearable devices to form a whole interactive system in 3D space. This kind of interactive system provides users with a more immersive experience and has the potential to be used in many application scenarios, such as sports training simulation, medical rehabilitation, entertainment and so on [172,174,189,190,191,192,193,194]. Apart from the software part which providing an actual interactive 3D environment, the integrated sensors collecting user’s motion, force, and tactile sensation information [195,196,197] also play an important role in the whole VR/AR interactive system. Chen et al. proposed a novel self-powered VR 3D-control sensor based on the triboelectric mechanism for controlling the attitude (both the position and rotation) of objects in 3D virtual space as shown in Figure 13a [172]. The two touching spheres as the sensing point with human fingers for 3D force information detecting and a “Virtual Assembly” application was successfully achieved. Combining triboelectric mechanism and wearable devices, a self-powered glove-based intuitive interface for diversified control applications in real/cyber space was presented by He et al. [174]. The demo of the virtual car’s movement control in VR space by this glove was shown in Figure 13b. All the sensory parts in their design were made of flexible and stretchable triboelectric materials, giving it high flexibility compared with rigid and bulky wearable human-machine interfaces. Similarly, a stretchable and transparent tactile sensor based on the hetero-contact microstructure was developed by Liao et al. [190]. These fingertip touch sensors characterized high sensitivity and fast response time and successfully converted fingertip events into visual control and interactive feedback in VR space (Figure 13c). Furthermore, Dejace et al. proposed a finger bending strain sensor based on stretchable gallium-based conductors and a virtual-reality scenario was demonstrated to accurately monitor human hand kinematics according to sensors’ resistance change as shown in Figure 13d [191]. These wearable human motion sensors, though capable of high flexibility and stretchability, can only collect limited sensory information due to their minimalist structural design. In order to establish more complex and immersive interactive systems in VR/AR space, many groups chose to combine different kinds of MEMS sensors and actuators together to implement more complicated functions, including human motion tracking, environment temperature simulation, haptic force feedback, and so on. Martínez et al. used an optical tracking system with light-emitting diode (LED) markers distributed on the glove to implement hand motion tracking and collision detection in VR space [192]. Virtual 3D geometric shapes can also be identified by adding 12 vibrotactile actuators which providing vibration feedback when contacting with virtual objects (Figure 13e). Lin et al. reported a method to find the suitable driving voltages of piezoelectric actuators with machine learning technology and gave users realistic continual lateral stroking and surface roughness tactile sensations in virtual environments [193]. A more complete and novel baseball pitch training system has been developed by Tsai et al. [194]. In their design, variable resistance was adopted for finger-bending sensation and the touching force was measured by setting up a pressure-sensitive resistor (PSR) on each fingertip. 3D positioning was achieved by a vive light-house as well as a wristband with IR receivers. Physical constraint and touch simulation for each finger can be achieved by servo motors and electric shocks. All of these sensors and actuators combined together to form a complete VR interactive system that allowed the player to gain practical experience with grasping a baseball in the virtual environment. With these commercialized MEMS sensors and actuators, it is not difficult to set up a VR/AR interactive system with multiple functions. However, how to combine these traditional rigid MEMS sensors/actuators with flexible NEMS wearable devices or replace them with NEMS wearable sensors to enhance the flexibility/stretchability of the VR/AR system as well as lowering system’s power consumption is still a hot topic and deserves to be researched in the future.

## 6. Outlooks and Conclusions

This review paper summarizes the development trends and perspectives of future sensors and MEMS/NEMS. Due to the fast development of 5G technology and IoTs, future sensors are facing new challenges and opportunities. Starting from the typical MEMS fabrication technologies, we selectively discussed the critical microfabrication technologies such as soft lithography, transfer-printing, and 3D packaging. Those engineering technologies in future sensors speeded up their commercial realization and improve the product’s stabilities. Toward a smaller dimension, i.e., NEMS, better sensing performance in sensors was easily obtained. For a better understanding of the MEMS/NEMS sensors, three typical kinds of MEMS sensors were introduced from the aspect of the operating frequency. With the help of the mechanical vibration [198] in the MEMS accelerometer and MEMS gyroscope, those sensors can be used as an energy harvesting device or the self-power sensor to identify its excitation acceleration. To decrease its power consumption, the idea of the zero-power or the wake-up was raised up interests by researchers, which meant the sensors with almost zero-power consumption (called accident-based or event-based sensor). Except for those above-mentioned technologies, the functional material is the other important factor that results in the development of the next-generation multi-functional sensor. With the improvement of living quality of human beings, MEMS acoustic sensors are becoming a hot research topic. However, the output signal from those acoustic sensors is on a small level and both with a high-level noise. To get across the output signal issues, machine learning was recently adopted to identify the physical meaning of the signals. Meanwhile, as to the gas identification in the MEMS sensor, the research trends were aiming at a small size, a high sensitivity, a low concentration sensing, a selectivity to different gases, and multi-gas sensing [199,200,201,202]. For example, with the help of 2D materials or MOFs [203,204], a low concentration and multi-gas sensing was recently reported in the area of human healthcare for the disease diagnose.

Toward the wearable sensing application, we observed the development trends from the conventional rigid silicon substrate sensor to the flexible sensor, i.e., the functional materials textile for wearable electronics application to extend our perceptions. The sensing functionalities of wearable electronics included but not limited to pressure, strain, electrophysiology, temperature, and blood oximetry. With the aid of multiple flexible devices across different anatomical locations, it is feasible to facilitate the development of the human body sensor network for human behavior identification. Furthermore, the rapid development of modern society has witnessed the daily increasing interaction between humans and machines, resulting in huge demands of smart human-machine interfaces. Around the big data for supersensitive sensing, a future sensor using machine learning dramatically promotes the development trend of next-generation intelligence sensing. Meanwhile, with the rapid development of the information industry and human-machine interface technology in recent years, VR or AR technology has become a hot research topic when combined with wearable devices to form a whole interactive system in 3D space. This kind of interactive system provides users with a more immersive experience in many application scenarios with the help of future sensors and MEMS/NEMS.

## Figures and Tables

**Table 1:** Comparison among MEMS acoustic sensor types.

MEMS Acoustic Sensor Type	Advantages	Disadvantages
Capacitive	High sensitivity High signal-to-noise ratio Low noise level	Sensitive to environment disturbances (moisture/dust/shock) Bias voltage required
Electret	High sensitivity Low power consumption	Sensitive to environment disturbances High charges required
Electromagnetic	Wide dynamic range Low noise level	Huge volume Low sensitivity
Piezoelectric	High acoustic overload Low power consumption High reliability	High noise level Low sensitivity
Optical	High sensitivity Wide dynamic range Immune to electromagnetic interference	External light source Fabrication and packaging difficulties

**Table 2:** Performance comparison in gas sensors.

Gas Sensor Type	Principle	Fabrication Method	Response Time	Target Gas	Working Temperature	Operation Concentration	Reference
SnO_2_ nanowire	Metal oxide	Local deposition	150 s	H_2_S	200 °C	20 ppm	[78]
Pd-Si naomesh	Particles	Nanosphere lithography	12 s	H_2_S NO_2_ CO	RT	50 ppm	[82]
Pd, SiNM	Diode	Lithography	22 s	H_2_	RT	0.5%	[84]
Metamaterial absorber	Mid-IR	Lithography	5 s	CO_2_	RT	40 ppm	[85]
ZIF-8	MOF	Solution-based	~300 s	CO H_2_ NO_2_	RT	10 ppm	[87]
Graphene	Band-gap	Laser	~120 s	H_2_	RT	600 ppm	[89]
